# Acute gastric dilatation and portal venous gas after pulsed field ablation for atrial fibrillation: a cautionary case report

**DOI:** 10.1093/ehjcr/ytag761

**Published:** 2026-10-22

**Authors:** Masato Sakaki, Ryuta Watanabe, Koichi Nagashima, Shu Hirata, Yasuo Okumura

**Affiliations:** Division of Cardiology, Department of Medicine, Nihon University School of Medicine, 30-1 Ohyaguchi-Kamicho, Itabashi-ku, Tokyo 173-8610, Japan; Division of Cardiology, Department of Medicine, Nihon University School of Medicine, 30-1 Ohyaguchi-Kamicho, Itabashi-ku, Tokyo 173-8610, Japan; Division of Cardiology, Department of Medicine, Nihon University School of Medicine, 30-1 Ohyaguchi-Kamicho, Itabashi-ku, Tokyo 173-8610, Japan; Division of Cardiology, Department of Medicine, Nihon University School of Medicine, 30-1 Ohyaguchi-Kamicho, Itabashi-ku, Tokyo 173-8610, Japan; Division of Cardiology, Department of Medicine, Nihon University School of Medicine, 30-1 Ohyaguchi-Kamicho, Itabashi-ku, Tokyo 173-8610, Japan

**Keywords:** Pulsed field ablation, Atrial fibrillation, Portal venous gas, Gastric dilatation, Vagal nerve injury, Case report

## Abstract

**Background:**

Pulsed field ablation (PFA) is considered to reduce collateral extracardiac injury compared with conventional thermal ablation. However, transient effects on autonomic nerves and adjacent smooth-muscle structures remain incompletely characterized.

**Case summary:**

An 80-year-old lean man underwent PFA for paroxysmal atrial fibrillation using a variable-loop circular catheter. Because stable tissue proximity was difficult to achieve around the left inferior pulmonary vein, additional ablation sets were delivered in this region. Approximately 24 h after the procedure, the patient developed acute abdominal pain. Computed tomography demonstrated marked gastric dilatation and portal venous gas with elevated lactate levels, without superior mesenteric artery occlusion or findings of irreversible bowel necrosis. Nasogastric decompression, fasting, intravenous fluids, and antibiotics resulted in rapid clinical and biochemical improvement. Portal venous gas resolved on follow-up imaging, oral intake was resumed, and the patient was discharged without surgical intervention.

**Discussion:**

The temporal relationship raises the possibility of a PFA-associated transient gastrointestinal dysmotility, although causality cannot be established from a single case. Potential mechanisms include transient effects on the periesophageal vagal plexus and/or adjacent smooth muscle. Portal venous gas was likely secondary to marked gastric dilatation rather than a direct effect of PFA.

Learning points

- PFA may rarely be temporally associated with transient gastrointestinal dysmotility despite its favourable collateral-tissue safety profile.
- Marked gastric dilatation can secondarily produce portal venous gas; prompt imaging and clinical assessment are essential to exclude irreversible intestinal ischaemia.
- When gastrointestinal symptoms occur after PFA, early recognition and gastric decompression may be important; routine prophylactic nasogastric tube placement cannot be recommended from a single case.

## Introduction

Pulsed field ablation (PFA) is a non-thermal energy source for catheter ablation of atrial fibrillation (AF). By inducing irreversible electroporation, PFA preferentially affects myocardial tissue and has shown a favourable collateral-tissue safety profile compared with thermal ablation techniques.^1–5^ Gastrointestinal dysmotility after AF ablation has traditionally been linked to periesophageal vagal injury during thermal ablation.^6,7^ Recent experimental observations indicate that PFA does not completely spare adjacent structures: transient non-transmural oesophageal injury and marked oesophageal contraction/retraction during PFA have been demonstrated, with subsequent healing.^8^ We report acute gastric dilatation with portal venous gas after PFA for AF and discuss potential autonomic and smooth-muscle mechanisms, although causality cannot be proven.

## Summary figure

**Table:** 

Time	Events
Day 0	PFA performed with 25 ablation sets, corresponding to 75 individual pulse deliveries, including 7 ablation sets at the left inferior pulmonary vein, corresponding to 21 pulse deliveries.
Post-ablation Day 1	Acute abdominal pain developed; CT showed marked gastric dilatation and portal venous gas.
Post-ablation Day 1	Nasogastric decompression, fasting, intravenous fluids, and antibiotics were initiated after surgical consultation.
Post-ablation Day 2	Portal venous gas resolved on follow-up CT; conservative management was continued.
Post-ablation Day 5	Nasogastric tube was removed, and oral intake was gradually resumed.
Post-ablation Day 17	The patient was discharged ambulatory without surgical intervention.

## Case presentation

An 80-year-old man with hypertension, diabetes mellitus, and chronic subdural haematoma underwent catheter ablation for paroxysmal AF. He was 163 cm tall and weighed 48.4 kg (body mass index, 18.2 kg/m^2^). Transthoracic echocardiography demonstrated preserved left ventricular systolic function with a left atrial volume index of 16.1 mL/m^2^.

The procedure was performed under conscious sedation with fentanyl (total dose, 125 μg), midazolam (total dose, 3 mg), and propofol (total dose, 80 mg). Atropine (0.5 mg) was also administered. Pulmonary vein isolation (PVI) was performed using the VARIPULSE variable-loop circular catheter with the TRUPULSE multichannel PFA generator and CARTO 3 mapping system (Biosense Webster, Inc., Irvine, CA, USA), with an irrigation flow of 30 mL/min. The standard PFA sequence delivered 880 V between adjacent electrode pairs and 1760 V between non-adjacent electrode pairs. One ablation set consisted of three 250-ms pulse deliveries separated by 10-s intervals. The total procedure time was 88 min. Catheter-tissue proximity was assessed using the tissue proximity indicator, and intracardiac echocardiography with a SOUNDSTAR catheter was used to reconstruct the left atrial geometry and integrate it with the preprocedural CT. A total of 25 ablation sets were delivered, corresponding to 75 individual pulse deliveries. At the left inferior pulmonary vein (LIPV), seven ablation sets were delivered, corresponding to 21 pulse deliveries, including four planned sets and three additional sets. Posterior wall isolation was not performed. Entrance and exit blocks were not assessed between the individual applications. After completion of all PFA applications, post-ablation mapping was performed using an OCTARAY catheter, and PVI was confirmed. No immediate procedural complications occurred (*Figure 1*).

**Figure 1:**
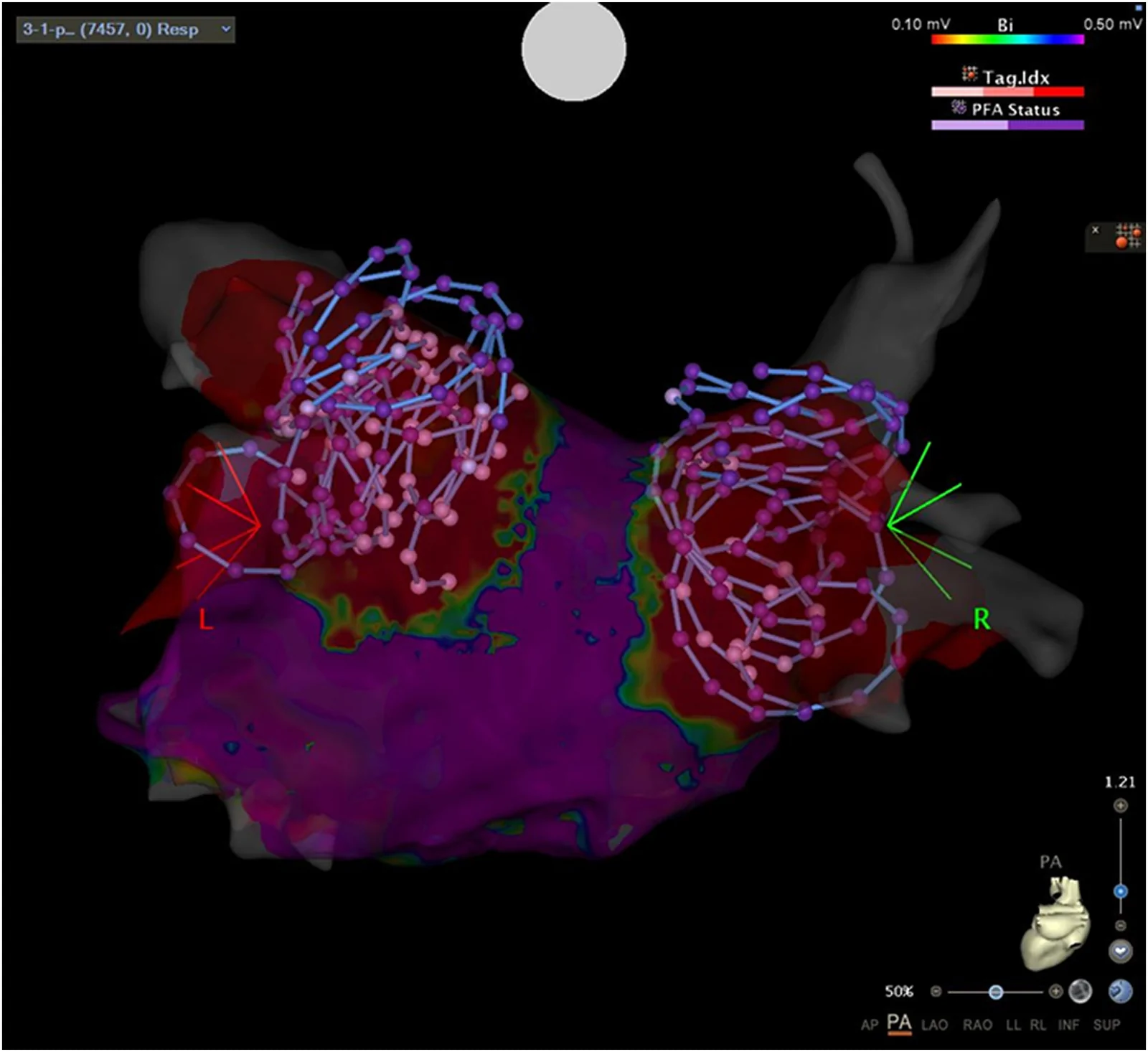
Pulsed field ablation lesion set and application sites. Electroanatomical map of the left atrium showing pulsed field ablation applications around the pulmonary vein antra and carinae. A total of 25 ablation sets, corresponding to 75 individual pulse deliveries, were delivered. At the left inferior pulmonary vein, seven ablation sets were delivered, corresponding to 21 pulse deliveries.

Approximately 24 h after the procedure, the patient developed acute abdominal pain. Physical examination revealed no rebound tenderness or guarding. Laboratory testing demonstrated elevated lactate levels of 5.1 mmol/L. Non-contrast computed tomography showed marked gastric dilatation and portal venous gas (*Figure 2*). Contrast-enhanced computed tomography demonstrated no superior mesenteric artery occlusion, bowel wall hypoenhancement, or pneumatosis intestinalis (*Figure 3*).

**Figure 2:**
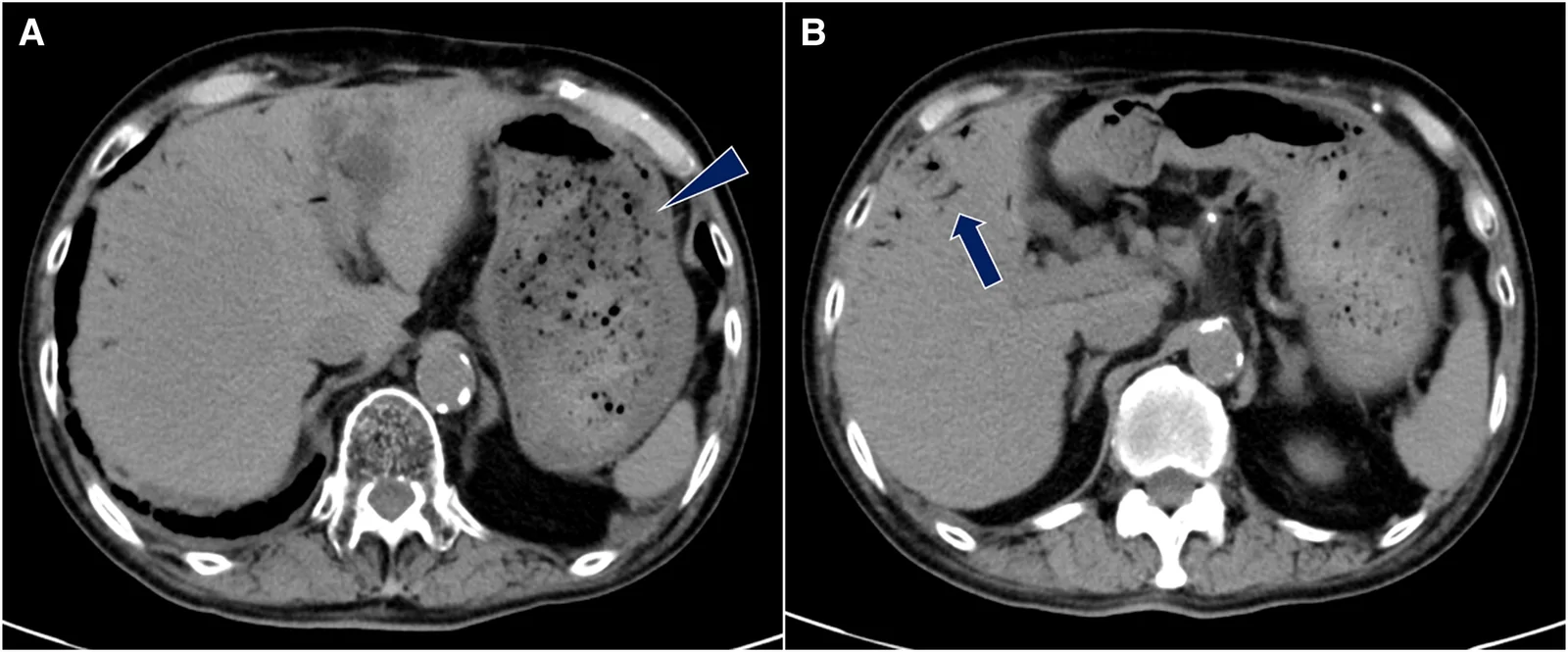
Acute gastric dilatation and portal venous gas after pulsed field ablation. Computed tomography obtained ∼24 h after ablation. (*A*) Marked gastric dilatation (arrowheads). (*B*) Portal venous gas within the intrahepatic portal venous system (arrows).

**Figure 3:**
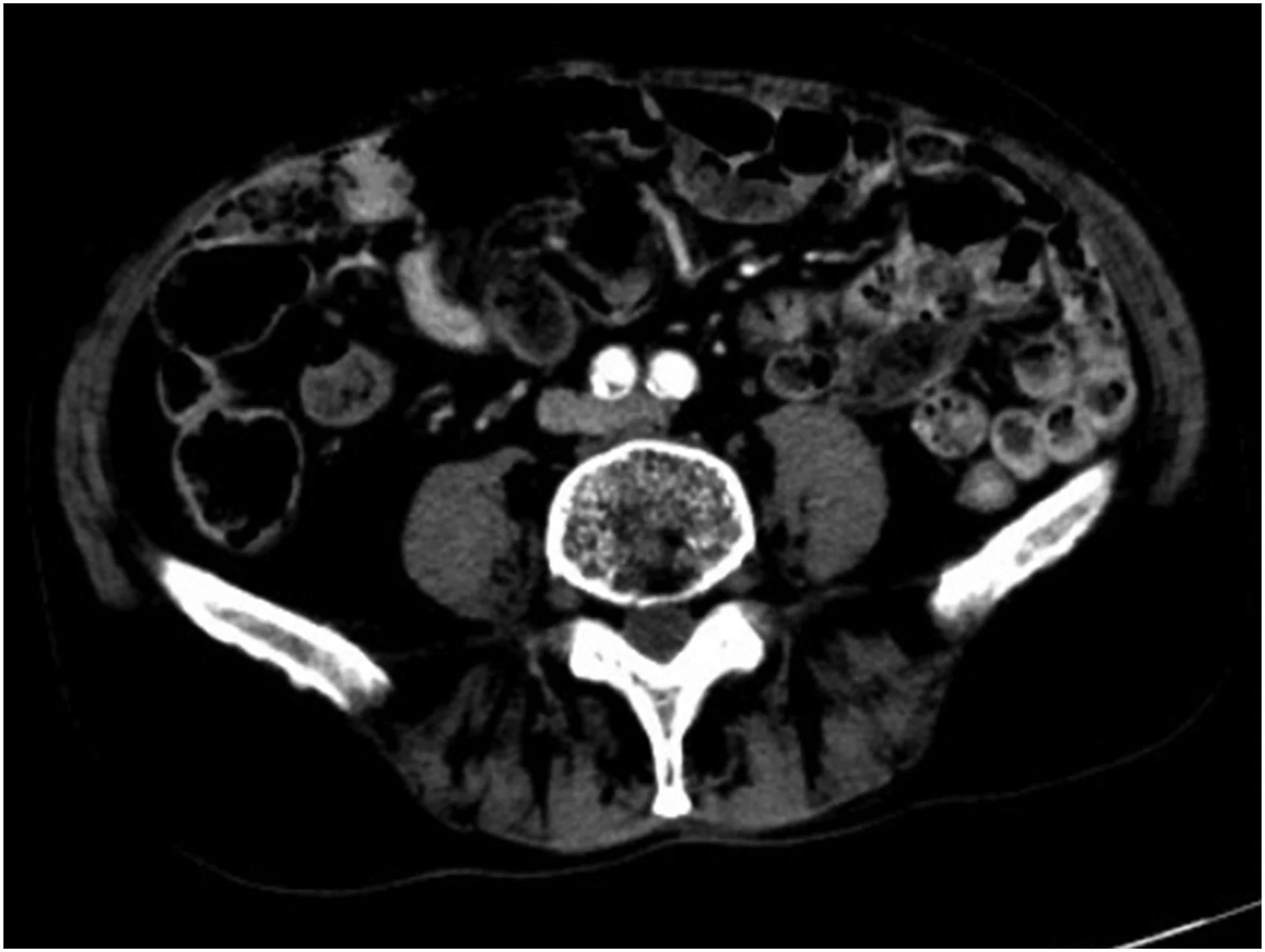
Contrast-enhanced computed tomography excluding irreversible intestinal ischaemia. Contrast-enhanced computed tomography showing preserved bowel wall enhancement without superior mesenteric artery occlusion or pneumatosis intestinalis.

After consultation with gastrointestinal surgeons, conservative treatment was initiated with nasogastric decompression, fasting, intravenous fluids, and cefmetazole. Lactate levels rapidly improved to 2.8 mmol/L within 2 h. Follow-up computed tomography on postoperative Day 2 demonstrated resolution of portal venous gas, although mild ileal ischaemic changes and ascites were observed (*Figure 4*). Because abdominal findings remained stable and lactate further improved, conservative management was continued with piperacillin–tazobactam. Although C-reactive protein increased to 23.5 mg/dL on postoperative Day 3, abdominal symptoms did not recur and lactate normalized. Oral intake was gradually resumed after removal of the nasogastric tube on postoperative Day 5. Follow-up imaging on postoperative Day 7 demonstrated improvement of intestinal findings. The patient was discharged ambulatory on postoperative Day 17 without recurrence of abdominal symptoms during hospitalization. At the 3-month outpatient follow-up, he remained free from recurrent abdominal symptoms and had no clinically apparent gastrointestinal sequelae.

**Figure 4:**
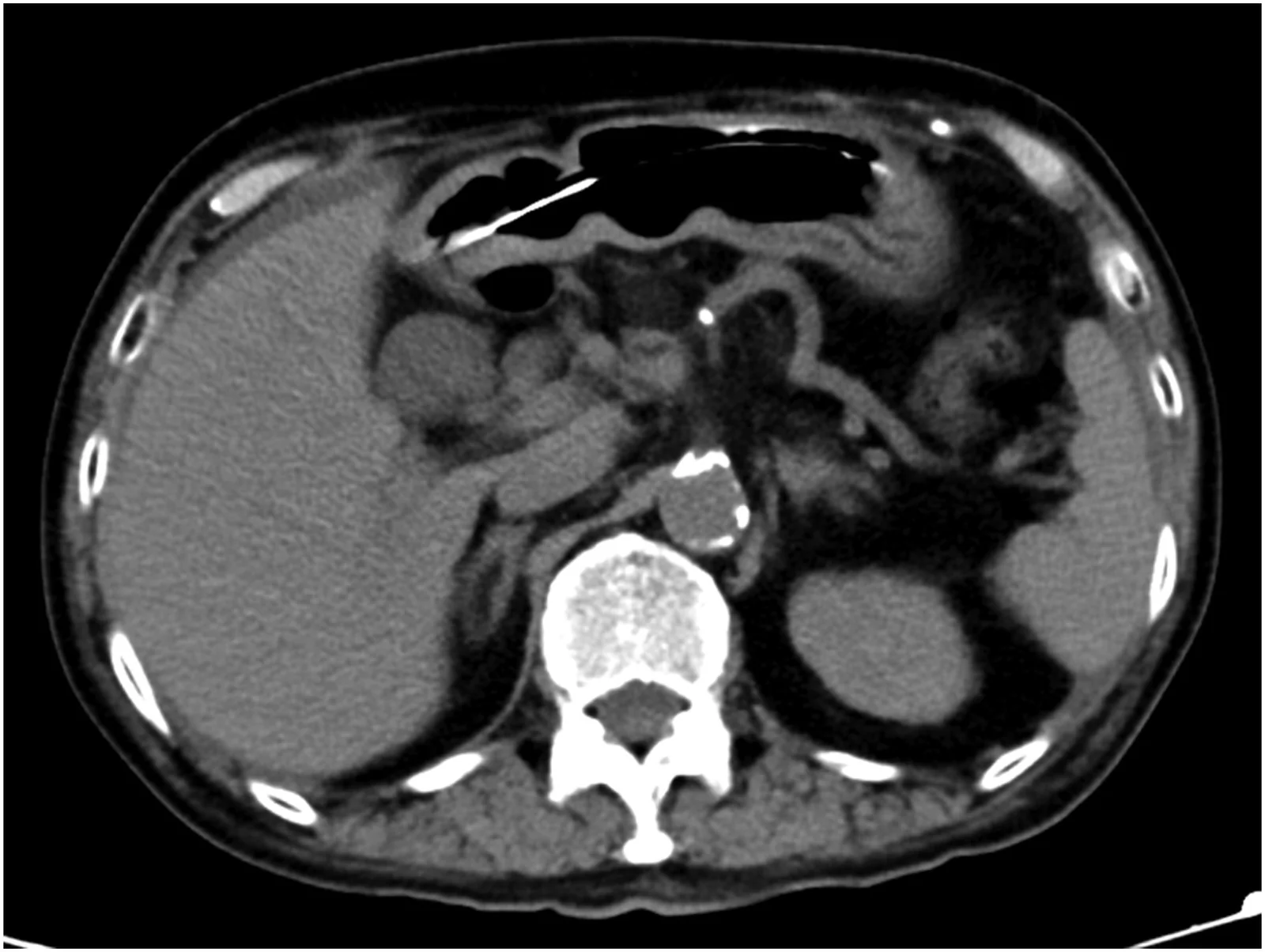
Resolution of portal venous gas following conservative treatment. Follow-up computed tomography on postoperative Day 2 demonstrating complete resolution of portal venous gas.

## Discussion

Acute gastric dilatation with portal venous gas occurred ∼24 h after PFA for AF. The temporal relationship makes a procedure-associated mechanism plausible, but causality cannot be established from a single case. Alternative pharmacological contributors, including sedative, opioid, and anticholinergic effects on gastrointestinal motility, cannot be excluded. The rapid response to gastric decompression and absence of major mesenteric arterial occlusion support reversible dysmotility rather than primary irreversible bowel ischaemia.

Gastrointestinal dysmotility after AF ablation has traditionally been attributed to injury of the periesophageal vagal plexus adjacent to the posterior left atrium.^6,7^ Although conventional thermal injury is unlikely after PFA, PFA should not be regarded as inert to all neighbouring tissues. Nies *et al*. recently demonstrated acute non-transmural oesophageal lesions after PFA that resolved by 14 days and observed consistent oesophageal contraction/retraction during atrial PFA across different catheter systems and waveforms.^8^ These findings suggest transient electroporation-related effects on smooth muscle and/or neural structures despite absent lasting structural injury. In the present patient, transient involvement of the periesophageal vagal network, gastric autonomic pathways, or adjacent smooth muscle therefore represents a biologically plausible, but unproven, explanation for the marked gastric hypomotility.

The procedural and anatomical setting may be relevant. The patient was lean and had a small left atrial volume, which may reduce the separation between the posterior left atrium and adjacent mediastinal structures. Stable tissue proximity around the LIPV was difficult to achieve, resulting in seven ablation sets at the LIPV, corresponding to 21 individual pulse deliveries, including three additional sets beyond the planned four sets. Repeated applications do not establish causality, and no gastrointestinal injury threshold has been defined; nevertheless, the high local application burden may have increased exposure of adjacent neural or smooth-muscle structures. From a procedural standpoint, avoiding unnecessary repeated ablation sets near the posterior LIPV may be prudent, particularly in lean patients with small left atrial anatomy. This possibility should be interpreted cautiously because the difficulty in maintaining tissue proximity could also imply less effective energy coupling during some applications.

Portal venous gas is unlikely to represent a direct PFA effect. Acute gastric dilatation is a recognized cause of portal venous gas: marked distension can increase intragastric pressure, compromise mucosal integrity and perfusion, and permit intraluminal gas to enter the portal venous circulation.^9^ This sequence is consistent with rapid resolution of portal venous gas after nasogastric decompression and supportive treatment. Thus, a reasonable mechanistic sequence is transient gastric dysmotility followed by severe gastric dilatation and secondary portal venous gas, rather than direct intravascular gas formation by PFA.

Routine prophylactic nasogastric tube placement during PFA cannot be recommended from a single case. However, unnecessary repeated ablation sets near the posterior LIPV should be avoided when possible, especially in lean patients with small left atrial anatomy, although this recommendation remains speculative. New abdominal pain, distension, nausea, or vomiting after PFA should prompt early evaluation for gastric dysmotility and other abdominal complications. When marked gastric dilatation is present, prompt nasogastric decompression may be appropriate, together with contrast-enhanced imaging and surgical consultation when portal venous gas, elevated lactate, or concern for intestinal ischaemia is present. These measures may distinguish reversible dysmotility from conditions requiring urgent surgery.

## Conclusion

We report a rare case of acute gastric dilatation with secondary portal venous gas after PFA for AF. Although causality cannot be proven, transient autonomic and/or smooth-muscle effects related to PFA are plausible mechanisms, particularly in the setting of repeated LIPV applications. Portal venous gas may arise secondarily from severe gastric distension and does not necessarily indicate irreversible intestinal necrosis. Early recognition, imaging, and gastric decompression may permit successful conservative management in appropriately selected patients.

## Patient perspective

The patient provided written informed consent for publication of this case report. No separate patient narrative was available.

## Lead author biography


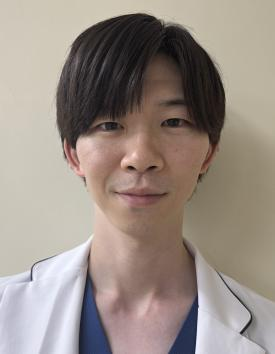


Masato Sakaki, MD, is a cardiology fellow at the Division of Cardiology, Department of Internal Medicine, Nihon University School of Medicine, Tokyo, Japan. His clinical and research interests include cardiac electrophysiology, catheter ablation for atrial fibrillation, and pulsed field ablation. He is actively involved in the management of complex arrhythmia patients and clinical research focused on improving the safety and efficacy of catheter-based therapies.

## Data Availability

The data underlying this article are available from the corresponding author upon reasonable request.
